# Reliability of clinically relevant 3D foot bone angles from quantitative computed tomography

**DOI:** 10.1186/1757-1146-6-38

**Published:** 2013-09-17

**Authors:** David J Gutekunst, Lu Liu, Tao Ju, Fred W Prior, David R Sinacore

**Affiliations:** 1 Applied Kinesiology Laboratory, Program in Physical Therapy, Washington University School of Medicine, St. Louis, MO 63108, USA; 2 Motion Analysis Laboratory, Department of Orthopedic Surgery, Mayo Clinic, Rochester, MN 55905, USA; 3 Department of Computer Science and Engineering, Washington University in St. Louis, MO 63105, USA; 4 Electronic Radiology Laboratory, Mallinckrodt Institute of Radiology, Washington University School of Medicine, St. Louis, MO 63108, USA

## Abstract

**Background:**

Surgical treatment and clinical management of foot pathology requires accurate, reliable assessment of foot deformities. Foot and ankle deformities are multi-planar and therefore difficult to quantify by standard radiographs. Three-dimensional (3D) imaging modalities have been used to define bone orientations using inertial axes based on bone shape, but these inertial axes can fail to mimic established bone angles used in orthopaedics and clinical biomechanics. To provide improved clinical relevance of 3D bone angles, we developed techniques to define bone axes using landmarks on quantitative computed tomography (QCT) bone surface meshes. We aimed to assess measurement precision of landmark-based, 3D bone-to-bone orientations of hind foot and lesser tarsal bones for expert raters and a template-based automated method.

**Methods:**

Two raters completed two repetitions each for twenty feet (10 right, 10 left), placing anatomic landmarks on the surfaces of calcaneus, talus, cuboid, and navicular. Landmarks were also recorded using the automated, template-based method. For each method, 3D bone axes were computed from landmark positions, and Cardan sequences produced sagittal, frontal, and transverse plane angles of bone-to-bone orientations. Angular reliability was assessed using intraclass correlation coefficients (ICCs) and the root mean square standard deviation (RMS-SD) for intra-rater and inter-rater precision, and rater versus automated agreement.

**Results:**

Intra- and inter-rater ICCs were generally high (≥ 0.80), and the ICCs for each rater compared to the automated method were similarly high. RMS-SD intra-rater precision ranged from 1.4 to 3.6° and 2.4 to 6.1°, respectively, for the two raters, which compares favorably to uni-planar radiographic precision. Greatest variability was in Navicular: Talus sagittal plane angle and Cuboid: Calcaneus frontal plane angle. Precision of the automated, atlas-based template method versus the raters was comparable to each rater’s internal precision.

**Conclusions:**

Intra- and inter-rater precision suggest that the landmark-based methods have adequate test-retest reliability for 3D assessment of foot deformities. Agreement of the automated, atlas-based method with the expert raters suggests that the automated method is a valid, time-saving technique for foot deformity assessment. These methods have the potential to improve diagnosis of foot and ankle pathologies by allowing multi-planar quantification of deformities.

## Background

Describing and quantifying foot deformities accurately and reliably is challenging to orthopaedic surgeons, podiatrists, and rehabilitation specialists. Bony deformities in the foot and ankle are multi-planar and therefore difficult to quantify by standard uni-planar radiographic measures. Much research has focused on developing and validating multi-segment foot and ankle models using optoelectronic motion capture based on skin-mounted reflective markers placed on palpable anatomic landmarks [1,2]. Three-dimensional (3D) imaging techniques such as magnetic resonance imaging (MRI) [3-5] and quantitative computed tomography (QCT) [6,7] have been used to quantify 3D bone-to-bone orientation angles *in vivo*.

**Figure 1:**
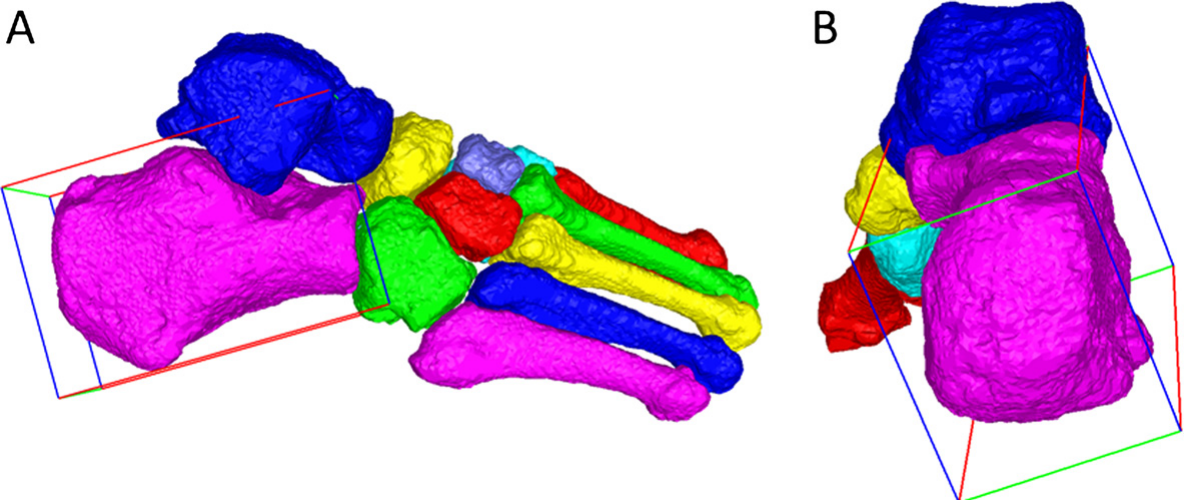
**Inertial axes reflecting bone shape.** Surface maps of the 7 tarsal and 5 metatarsal bones, with a minimum bounding volume surrounding calcaneus representing the direction vectors for the principal inertial axis (red), second inertial axis (green), and third inertial axis (blue). **(A)** Lateral view of an exemplar foot, showing that the principal inertial axis reasonably approximates calcaneal pitch; **(B)** Posterior view of the foot, showing that the second and third inertial axes fail to align with clinically-relevant axes representing the local medial-lateral and inferior-superior axes of calcaneus.

These 3D imaging studies use the principal components method to define bone coordinate axes, meaning that the bone orientation axes reflect solely the bones’ shapes. While these inertial axes mimic clinical definitions of bone axes for long bones such as the metatarsals and phalanges, inertial axes may fail to align with clinical bone axes for the tarsals, particularly the lesser tarsals (cuboid and navicular) and the hind foot bones (calcaneus and talus). For example, while the primary inertial axis for calcaneus (red direction vectors in Figure 1A) approximates the measured angle of calcaneal pitch on a lateral X-ray, the second and third inertial axes (green and blue direction vectors in Figure 1B) provide inaccurate representations of the desired medial-lateral (green) and superior-inferior (blue) axes that are used to characterise frontal plane and transverse plane deformities.

Others have proposed anatomy-based axes to track morphological differences in the subtalar and talocrural joints [8], but to our knowledge, no previous research has described methods and established bone-to-bone angular reliability for *in vivo* imaging using clinically relevant definitions of foot bone axes. As an initial assessment of the feasibility of a novel method to measure 3D bone orientation angles, we elected to focus on the lesser tarsals and hind foot bones, for four main reasons. First, the hind foot and lesser tarsal bones present the most pressing need for clinically-defined bone axes to replace inertial axes. Second, these bones are frequently involved in foot deformities, and influence arch height and foot function. Third, subtalar and midtarsal joint deformities frequently lead to mal-alignments in the midtarsal joints and in hind foot:forefoot coupling [9]. Finally, pilot testing of our landmark-based methods suggested that angular measurements of hind foot and lesser tarsal bones were least reliable, both within and between raters, largely because mimicking clinically-based orientation axes used in radiographic measures [10] necessitates identifying bone surface features that are not always located at bony edges. For example, replicating lateral X-ray measures of talo-calcaneal angle or Meary’s angle [11] requires placing a landmark on the curved surfaces of calcaneus and talus, which can be challenging to reliably replicate on surface reconstructions from QCT.

Thus, the purposes of this study were to: (i) describe a non-invasive anatomic landmark-based method of defining 3D bone orientation axes for foot bones using bone atlases derived from segmented QCT surface images; (ii) determine intra-rater and inter-rater reliability and angular precision for select hind foot and lesser tarsal angles; and (iii) assess the agreement between expert raters and a template-based automated method of placing anatomic landmarks.

## Methods

### Participants

Ten healthy participants (5 females, 5 males; age 26.6 ± 3.8 years; height 174.1 ± 8.5 cm; mass 76.5 ± 15.8 kg) with no known foot pathology underwent bilateral QCT scans of the foot and ankle. All participants read and signed an informed consent document outlining the research protocol and associated risks and benefits. The research protocol (IRB ID# 201103036) was approved by the institutional review board at Washington University School of Medicine in Saint Louis, Missouri, USA.

### QCT processing and bone atlases

The 7 tarsal and 5 metatarsal bones were segmented from surrounding soft tissues and from each other using ImageJ filtering plug-ins, Analyze® software (Biomedical Imaging Resource, Mayo Clinic, Rochester, MN), and custom semi-automated graph cut software [12-15]. The end result of segmentation is a set of binary, filled object maps which define the QCT voxel coordinates for each bone.

The segmented object maps and grayscale voxel data were imported into a custom Bone Measurement Tool (BMT), a fully automated pipeline for registering tarsal and metatarsal bones to a pre-defined foot bone atlas [12,15]. The *atlas* is made up of template bone surfaces, one for each tarsal and metatarsal, and a control grid around each template bone. The template bone surfaces are constructed from the QCT scan of a healthy subject. The control grids, defined using a geometric structure known as subdivision mesh, are used to warp the template bone surface geometrically with the goal of matching a test bone surface. *Registration*, the process of fitting the atlas to a test foot, starts with aligning each template bone in the atlas to the corresponding test bone using whole-bone rotation, scaling and translation, followed by a local warping that accounts for the fine-scale anatomical differences between the test and template bones [14]. Atlas registration provides a “mapping” between points on the surface (or in the interior) of a template bone and those on the test bone. The BMT was originally used to assess bone mineral density for foot bones, which utilises the interior mapping offered by atlas registration [12-14]. In the current study, BMT was used to establish correspondence between the surfaces of the test and the template bones, with the goal of mapping landmark locations from the template bones to the test bones. To this end, BMT was expanded beyond the task of computing bone mineral density, to incorporate two different methods to locate bone surface landmarks that are then used to define bone spatial orientation:

*Method 1:* A graphical user interface that allows the rater to rotate segmented foot bones – either alone or as a group of bones – and record the positions of anatomical landmarks on the bone surface meshes.

*Method 2:* An automated landmark placement function, in which the user provides landmark locations only on template bone surfaces in the atlas. Using automated atlas registration, these landmarks are automatically located and recorded on every test foot in an entire data set.

In this study, we used Method 1 to assess intra-rater and inter-rater precision for manual placement of anatomical landmarks, and also compared the results from Method 1 to the automated Method 2.

### Selection of anatomic landmarks and bone orientation axes

**Figure 2:**
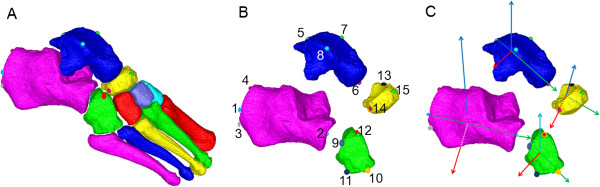
**Atlas-based QCT surface maps.** Surface maps show **(A)** all 7 tarsal and 5 metatarsal bones; **(B)** expanded view of hind foot and lesser tarsal bones with labeled anatomic landmarks; **(C)** bone orientation axes derived from anatomical landmarks. For each bone, the X-axis is shown in red, Y-axis is green, and Z-axis is blue.

Each bone’s 3D axes were defined based on the locations of 3–4 anatomic landmarks (Table 1). Landmarks were chosen by expert consensus to ensure clinical relevance and consistency with established planar bone axis designations from the orthopaedics literature [16,17] to produce clinically relevant bone axes. Definitions of bone axes based on landmark positions are shown in Table 2. Bone axis directions followed the convention of the Oxford Foot Model used in multi-segment foot kinematics [1], with the resulting + X axis for each bone directed roughly to the right (medial for left feet and lateral for right feet), +Y pointing anteriorly (roughly axial for calcaneus, talus, and cuboid), and + Z directed in a quasi-vertical direction. For all bones except navicular, the Y axis was the first axis defined, as the unit vector connecting two anatomical landmarks representing the proximal and distal termini of the main longitudinal axis of the bone. Due to the shape of navicular, X axis (medial-lateral) was defined first as this represents the longest bone dimension. For all bones, the second axis was defined as the cross-product of the first axis and a temporary (‘dummy’) axis, and the third axis was defined by crossing the first and second axes. Bone axis computations for each bone are provided in Table 2, and a schematic showing landmark placement and bone axes is shown in Figure 2.

**Table 1:** Anatomic landmarks and marker placement precision

**Bone**	**Landmarks**	**Description**
**Calcaneus**	1. Posterior calcaneus	Midpoint of posterior surface of calcaneal tuberosity, centred both medial-laterally and vertically.
	2.Anterior calcaneus	Centre of the anterior surface of calcaneus, where calcaneus articulates with cuboid.
	3.Inferior calcaneus	Medial-lateral midline of posterior surface of calcaneal tuberosity, along the inferior border.
	4.Superior calcaneus	Medial-lateral midline of posterior surface of calcaneal tuberosity, along the superior border.
**Talus**	5. Posterior talus	At medial-lateral midline of the posterior aspect of talus.
	6. Anterior talus	Centre of convex surface of talar head, centred both medial-laterally and vertically.
	7. Medial talus	Dorsal maximum of the medial edge of talar trochlea articular surface.
	8. Lateral talus	Dorsal maximum of the lateral edge of the talar trochlea articular surface.
**Cuboid**	9. Posterior cuboid	Centre of proximal cuboid articular surface (articulation with calcaneus).
	10. Anterior cuboid	Centre of distal cuboid articular surface (articulation with fourth and fifth metatarsals).
	11. Inferior cuboid	Inferior-lateral edge of the tuberosity of cuboid.
	12. Superior cuboid	Most superior, dorsal “point” of cuboid.
**Navicular**	13. Medial navicular	Medial aspect of navicular, centred in the anterior-posterior direction.
	14. Lateral navicular	Lateral aspect of navicular; centred in the anterior-poster direction.
	15. Superior navicular	Superior surface of navicular; at the most superior point of its dorsal surface.

**Table 2:** Bone orientation definitions based on anatomical landmarks (right foot)

**Bone**	**First axis**	**Temporary axis**	**Second axis**	**Third axis**
**Calcaneus**	**Y**_**calc **_**= |** 1 → 2 **|**	**t**_**calc **_**= |** 3 → 4 **|**	**X**_**calc **_**= Y**_**calc**_ X **t**_**calc**_	**Z**_**calc **_**= X**_**calc**_ X **Y**_**calc**_
**Talus**	**Y**_**tal **_**= |** 5 → 6 **|**	**t**_**tal **_**= |** 7 → 8 **|**	**Z**_**tal **_**= t**_**tal**_ X **Y**_**tal**_	**X**_**tal **_**= Y**_**tal**_ X **Z**_**tal**_
**Cuboid**	**Y**_**cub **_**= |** 9 → 10 **|**	**t**_**cub **_**= |** 11 → 12 **|**	**X**_**cub **_**= Y**_**cub**_ X **t**_**cub**_	**Z**_**cub **_**= X**_**cub**_ X **Y**_**cub**_
**Navicular**	**X**_**nav **_**= |** 13 → 14 **|**	**t**_**nav **_**= |** 13 → 15 **|**	**Y**_**nav **_**= t**_**nav**_ X **X**_**nav**_	**Z**_**nav **_**= X**_**nav**_ X **Y**_**nav**_

Two expert raters completed two repetitions each for twenty feet, placing anatomic landmarks on the atlas-derived surfaces of calcaneus, talus, cuboid, and navicular. Raters were blinded to the participant number for each foot: image files were de-identified and reassigned a randomly generated file number, then presented in a randomised order. Rater 1 is a biomedical engineer who was a Ph.D. candidate at the time of data collection, with 8 years of research experience in musculoskeletal biomechanics, including 2 years working with QCT image data. Rater 1 also beta-tested early versions of the BMT, which entailed substantial time (>300 hrs) manipulating 3D bone images. Rater 2 is a physical therapist and research scientist with 20 years of experience specialising in foot and ankle complications of diabetes, including more than 15 years working with foot and ankle X-rays, and 2 years working with QCT image data.

Landmarks were also recorded using the automated method based on a landmark template embedded within the bone atlases [15]. Cardan rotation sequences (XY’Z”) of bone axes were used to produce sagittal (α), frontal (β), and transverse (γ) plane angles of the cuboid with respect to the calcaneus (Cub:Calc), talus with respect to the calcaneus (Tal:Calc), navicular with respect to the talus (Nav:Tal), and navicular with respect to calcaneus (Nav:Calc). Other research groups have used a Cardan rotation sequence of sagittal, transverse, and frontal plane angles for the hind foot bones [6,18], but we chose a Cardan rotation sequence of sagittal, frontal, and transverse to be consistent with multi-segment kinematics foot models based on the Oxford Foot Model [1]. Moreover, the sagittal, frontal, transverse rotation sequence is consistent with the convention for ankle angles recommended by the International Society of Biomechanics [19].

### Angular reliability assessment

We assessed three measures of reliability: intra-class correlation coefficient (ICC), angular precision, and agreement. The ICC is a relative measure of reliability that reflects the test-retest consistency of a measurement [20]. The operational definition of intra- and inter-rater *precision* follows guidelines put forth by the International Society of Clinical Densitometry, which describe precision as “the ability of a quantitative measurement technique to reproduce the same numerical result when repeatedly performed in an identical fashion” [21]. We use the term *agreement* to describe the relationship between the manual and automated landmark placement data, as the techniques are not identical.

### Data analysis

A two-way, random effects, single-measures ICC model (ICC 2,1) [20] was used. For the inter-rater ICC calculations – Rater 1 versus Rater 2 and each rater versus the automated method – angles were computed using average landmark positions from the two trials. As the automated method results in identical landmarks for repeat measures of the same foot, no averaging was completed.

Intra-rater precision, inter-rater precision, and agreement for each rater versus the automated method were calculated for each bone-to-bone angular rotation as the root mean square standard deviations (RMS-SD). Whereas the ICC assesses the relative consistency of a measure, RMS-SD assesses the precision and absolute consistency. To compute RMS-SD, first the standard deviation (SD) is calculated across repeat angular measurements repeated trials for each of the 20 feet. For intra-rater precision, SD values represent variability across repeated trials by the same rater; for inter-rater precision, SD values represent variability across the two raters. The SD values are then squared, and these squared SD values are summed across all samples, divided by *N* to result in the mean squared SD. The final step is to compute the square root of the mean squared SD, resulting in the RMS-SD:

$$\mathrm{RMS}-\mathrm{SD}=\sqrt{\frac{\sum SD^{2}}{N}}$$

The RMS-SD is recommended by the International Society for Clinical Densitometry [21], and has been used to assess angular precision in X-ray measures of the foot [9].

One advantage of the RMS-SD is that it represents the expected variability for repeat measures of the same angle, expressed in the units of measurement [21]. The RMS-SD is a measure of the variability of bone angles at the level of the individual participant – *i.e.,* the number of degrees that an individual’s bone angle would vary due to measurement error. In order to assess the repeatability at the level of all participants, we also report intraclass correlation coefficients (ICCs) for intra-rater precision, inter-rater precision, and agreement between raters and the automated method [20].

## Results

ICC results were generally moderate to high. The majority of intra-rater ICC values for Rater 1 were ≥ 0.90, though several sagittal plane (α) angles had low ICC values (Table 3), including Tal:Calc and Nav:Tal. Similarly, most intra-rater ICC values for Rater 2 were moderate to high, with the exception of sagittal plane angles. The inter-tester ICC values of Rater 1 versus the automated method exceeded inter-tester ICC values for Rater 1 versus Rater 2. Inter-tester ICC values of Rater 2 versus the automated method were comparable to the intra-tester ICC values for Rater 2.

**Table 3:** Intraclass correlation coefficients for bone-to-bone angles

	**Intra-rater intraclass correlation coefficient**		**Inter-rater intraclass correlation coefficient**		
	**Rater 1**	**Rater 2**	**Rater 1 *vs. *Rater 2**	**Rater 1 *vs. *Auto**	**Rater 2 *vs. *Auto**
***Cuboid:Calcaneus***					
Sagittal plane (α)	0.77 (0.51, 0.90)	0.51 (0.10, 0.77)	0.68 (0.35, 0.86)	0.72 (0.42, 0.88)	0.63 (0.27, 0.84)
Frontal plane (β)	0.87 (0.71, 0.95)	0.65 (0.31, 0.85)	0.59 (0.21, 0.82)	0.63 (0.27, 0.84)	0.41 (0.03, 0.72)
Transverse plane (γ)	0.95 (0.87, 0.98)	0.75 (0.47, 0.89)	0.93 (0.83, 0.97)	0.90 (0.76, 0.96)	0.81 (0.57, 0.92)
***Talus:Calcaneus***					
Sagittal plane (α)	0.43 (0.01, 0.73)	0.17 (−0.28, 0.56)	0.56 (0.17, 0.80)	0.50 (0.08, 0.77)	0.24 (−0.21, 0.61)
Frontal plane (β)	0.94 (0.84, 0.97)	0.85 (0.66, 0.94)	0.84 (0.65, 0.94)	0.78 (0.52, 0.91)	0.82 (0.59, 0.92)
Transverse plane (γ)	0.98 (0.96, 0.99)	0.96 (0.90, 0.98)	0.98 (0.97, 0.99)	0.95 (0.88, 0.98)	0.93 (0.84, 0.97)
***Navicular:Talus***					
Sagittal plane (α)	0.35 (0.10, 0.68)	0.61 (0.23, 0.82)	0.39 (−0.06, 0.70)	0.41 (0.03, 0.72)	0.48 (0.06, 0.75)
Frontal plane (β)	0.95 (0.89, 0.98)	0.57 (0.18, 0.80)	0.57 (0.19, 0.81)	0.99 (0.97, 1.00)	0.59 (0.21, 0.81)
Transverse plane (γ)	0.93 (0.84, 0.97)	0.80 (0.56, 0.91)	0.86 (0.69, 0.94)	0.85 (0.65, 0.94)	0.62 (0.26, 0.83)
***Navicular:Calcaneus***					
Sagittal plane (α)	0.71 (0.39, 0.87)	0.42 (0.01, 0.72)	0.61 (0.24, 0.82)	0.71 (0.40, 0.87)	0.75 (0.47, 0.89)
Frontal plane (β)	0.98 (0.96, 0.99)	0.86 (0.67, 0.94)	0.92 (0.80, 0.97)	0.97 (0.93, 0.99)	0.89 (0.73, 0.95)
Transverse plane (γ)	0.97 (0.92, 0.99)	0.87 (0.69, 0.95)	0.92 (0.81, 0.97)	0.95 (0.87, 0.98)	0.85 (0.65, 0.94)

Intraclass correlation coefficients (95% confidence intervals) in sagittal (α), frontal (β), and transverse (γ) bone-to-bone orientation angles for manual and automated anatomical landmark placement.

Across sagittal, frontal, and transverse angles for the four bone-to-bone orientations analysed, Rater 1 had lower RMS-SD than Rater 2 (Table 4). Averaged across all planes and bone-to-bone orientations, intra-rater precision averaged 2.3° for Rater 1 and 4.1° for Rater 2. The single highest intra-rater RMS-SD for Rater 2 (Cub:Calc frontal plane angle) was 6.1°, and the highest average angular RMS-SD (for Nav:Calc) was 4.9°. Inter-rater precision was slightly lower than the intra-rater precision for Rater 2, with highest inter-rater variability in the frontal plane angle of Cub:Calc (7.1° RMS-SD) and the frontal plane angle of Nav:Tal (6.1° RMS-SD). For both raters, precision values between manual placement of landmarks and the template-based automated method were comparable to intra-rater precision. The average RMS-SD values between Rater 1 and the automated method (2.7°) were lower than the average RMS-SD values between Rater 1 and Rater 2 (3.7°).

**Table 4:** Intra- and Inter-rater angular precision for bone-to-bone angles

	**Intra-rater precision**		**Inter-rater precision**	**Agreement with automated method**	
	**Rater 1**	**Rater 2**	**Rater 1 *vs. *Rater 2**	**Rater 1 *vs. *Auto**	**Rater 2 *vs. *Auto**
***Cuboid:Calcaneus***					
Sagittal plane (α)	1.9°	3.4°	2.0°	2.1°	2.4°
Frontal plane (β)	2.9°	6.1°	7.1°	4.4°	6.4°
Transverse plane (γ)	1.7°	2.7°	1.6°	2.9°	3.9°
***Talus:Calcaneus***					
Sagittal plane (α)	3.3°	3.7°	4.4°	2.3°	5.9°
Frontal plane (β)	1.9°	3.1°	2.7°	3.0°	3.3°
Transverse plane (γ)	1.4°	2.4°	1.5°	2.7°	2.9°
***Navicular:Talus***					
Sagittal plane (α)	3.6°	5.7°	3.5°	3.5°	3.3°
Frontal plane (β)	2.3°	4.7°	6.1°	1.3°	6.2°
Transverse plane (γ)	1.6°	2.9°	2.4°	2.4°	3.8°
***Navicular:Calcaneus***					
Sagittal plane (α)	2.4°	5.9°	5.1°	2.3°	4.9°
Frontal plane (β)	2.7°	5.6°	4.9°	3.3°	6.0°
Transverse plane (γ)	2.1°	3.1°	2.8°	2.7°	3.8°

Root-mean square standard deviations, in degrees, in the sagittal (α), frontal (β), and transverse (γ) bone-to-bone orientation angles for manual and automated anatomical landmark placement.

## Discussion

We have reported 3D angular precision for two expert raters and an automated template method to determine clinically relevant bone orientations from bone atlases based on segmented QCT scans of the foot. In contrast to previous 3D imaging methods to define foot bone orientation axes using the shape-dependent bone inertial axes [3,6], the methods presented here define 3D bone axes using anatomical landmarks, which provides added clinical relevance.

In general, most ICCs indicated moderate or high reliability within raters, between raters, and comparing each rater to the automated method. Notably, the sagittal plane (α) angles exhibited the lowest overall ICC values, especially for Talus:Calcaneus and Navicular:Talus bone-to-bone orientations. These results reflect the difficulty locating landmarks on the talus, particularly the posterior talus (landmark 5 in Figure 2). Additionally, sagittal plane angles had low ICC values partly because these angles had less variability across individual participants. If there is little between-subjects variability, then ICCs will be low even though trial-to-trial variability is small. Conversely, if individual participants’ values differ widely from each other, then ICC values will be high even if trial-to-trial variability is large [20].

Intra-rater angular precision averaged 2.3° and 4.1° for the two expert raters, and inter-rater angular precision averaged 3.7°. Thus, the present study suggests that atlas-based automated landmark methods can replicate landmark locations with equivalent precision as an expert rater. Moreover, these angular precision results are comparable to uni-planar radiographic precision [10] and have the advantage of providing a full 3D representation of static bone-to-bone angles, especially in the frontal plane and for bone-to-bone angles that are obscured during planar X-rays.

To achieve the level of intra-rater and inter-rater precision observed in this study, the raters required roughly 6 to 8 minutes *per bone* in order to manipulate bone surface maps within the graphical user interface and place landmarks. The automated method results in equivalent angular precision with negligible processing time, thus an additional advantage of the atlas-based automated method is a significant time savings.

The variability observed in bone-to-bone orientation angles can likely be improved by assessing which individual landmarks had the highest spatial variability [18]. Results from the present study would suggest that the atlas-based automated landmark method can replicate landmarks with equivalent or superior precision as an expert rater, and future studies are readily adaptable to using different definitions of bone axes, especially if a landmark position with higher test-retest precision can be adopted. A possible long-term improvement may follow techniques of optoelectronic motion capture by choosing the most reliable anatomical landmarks, then creating virtual landmarks to define bone axes in the most clinically relevant manner. Doing so could maximise both angular precision and clinical relevance. For example, in motion capture, several bony landmarks on the tibia (tibial tuberosity, medial malleolus) and fibula (fibular head, lateral malleolus) are easily palpable, but in order to create the most clinically relevant axes for the shank segment, it is preferable to define direction vectors based on virtual landmarks, such as the midpoint between the medial and lateral malleoli to define the distal endpoint of the shank segment.

One potential limitation of the present study is that the 3D bone-to-bone orientation methods utilise QCT, which imparts a low amount of radiation to the participant. Future research may replicate these methods using non-radiating 3D imaging modalities such as MRI, which would help extend the technique’s utility to pediatric populations with foot deformities, such as club foot in individuals with cerebral palsy. To expand future applications of the BMT software, it will be made available for research purposes upon request. Additionally, we envision future application of these methods to clinical populations who experience foot and ankle deformity, such as rheumatoid arthritis, diabetic neuropathy (including Charcot neuropathic osteoarthropathy), and Charcot-Marie-Tooth disease, or to provide clinically relevant definitions of bone axes to use in concert with bone motion studies using bone pins [22-24] or multi-plane fluoroscopy [25].

## Conclusions

We have developed novel methods to assess static, 3D foot bone-to-bone orientation angles using clinically relevant bone axes derived from QCT bone surface meshes. Generally moderate to high ICC values and high intra- and inter-rater precision suggest that the methods presented here are reliable for 3D assessment of foot deformities. Equivalent precision of a template-based automated method compared to expert raters suggests that the automated method may offer time savings that will enhance clinical applicability. These methods may be used in a variety of clinical populations to aid diagnosis and classification of foot and ankle pathologies.

## Competing interests

For this manuscript, “Atlas-based, clinically relevant three-dimensional foot bone-to-bone orientation angles derived from quantitative computed tomography” all authors state that they have no financial or personal conflicts of interest that could influence the submitted work.

## Authors’ contributions

DJG provided concept, data collection, data analysis, research design, writing, critical review, and revision for this manuscript. LL provided data analysis software, critical review, and revisions for this manuscript. TJ provided data analysis software, writing, critical review, and revisions for this manuscript. FWP provided concept, funding support, critical review, and revision for this study and manuscript. DRS provided concept, data collection, data analysis, research design, writing, funding support, critical review, and revision for this study and manuscript. All authors read and approved the final manuscript.
